# Antiarrhythmic effect of sevoflurane as an additive to HTK solution on reperfusion arrhythmias induced by hypothermia and ischaemia is associated with the phosphorylation of connexin 43 at serine 368

**DOI:** 10.1186/s12871-018-0656-8

**Published:** 2019-01-08

**Authors:** Wei Chao Li, Hong Gao, Ju Gao, Zi Jun Wang

**Affiliations:** 1 Department of Anesthesiology, The Affiliated Hospital of Guizhou Medical University, Guiyang, Guizhou, China; 2 Department of Anaesthesiology, North Jiangsu People’s Hospital, Yangzhou University, Yangzhou, China

**Keywords:** Sevoflurane, Gap junction remodelling, Cx43 phosphorylation at Ser368, Reperfusion ventricular arrhythmia, Hypothermic ischaemic storage

## Abstract

**Background:**

Reperfusion ventricular arrhythmia (RA) associated with hypothermic ischaemic storage is increasingly recognized as a substantial contributor to adverse consequences after heart transplantation. Ischemia- or hypothermia-induced gap junction (GJ) remodelling is closely linked to RA. Reducing GJ remodelling contributes to RA attenuation and is important in heart transplantation. However, sevoflurane has an antiarrhythmic effect associated with the connexin 43 (Cx43) protein that has not yet been fully established.

**Methods:**

Hearts were divided into two groups according to a random number table: all hearts were arrested by an infusion of histidine-tryptophan-ketoglutarate (HTK) solution (4 °C) followed by (1) storage in HTK solution (4 °C) alone for 6 h (*n* = 8, Control group) or (2) storage in HTK solution supplemented with sevoflurane (2.5%) (4 °C) for 6 h (n = 8, Sevo-HTK group). First, the total Cx43 level and the phosphorylation of Cx43 at Ser368 (Cx43-pS368) were assessed by Western blotting, and the distribution of Cx43 was assessed by immunohistochemistry. Second, programmed electrical stimulation (PES) and monophasic action potential (MAP) recording were used to analyse the MAP duration (MAPD), conduction velocity (CV) and transmural repolarization dispersion (TDR). In addition, haematoxylin and eosin (HE) and terminal deoxynucleotidyl transferase-dUTP nick end labelling (TUNEL) staining were individually used to investigate the degree of myocardial pathological damage and cell apoptosis. Finally, bipolar electrograms were used to record the graft re-beating time and monitor RA during reperfusion for 15 to 30 min.

**Results:**

Sevo-HTK solution relatively increased the total Cx43 (*P* < 0.01) and Cx43-pS368 (P < 0.01) levels and prevented Cx43 redistribution (*P* < 0.05) and CV slowing (*P* < 0.001) but did not change TDR (*P* > 0.05). Additionally, the Cx43-pS368/total Cx43 ratio (P>0.05) was similar in the two groups. However, with Sevo-HTK solution, the graft re-beating times were shortened, myocardial pathological damage was ameliorated, and the number of apoptotic cells was markedly decreased.

**Conclusion:**

The reduction in hypothermia and ischaemia-induced reperfusion arrhythmias by the addition of sevoflurane to HTK solution may be related to the phosphorylation of Cx43 at serine 368.

## Background

Hypothermic ischaemic storage at 4 °C has been the most widely used technique for preserving retrieved hearts [1]. Reperfusion ventricular arrhythmia (RA) associated with hypothermia-ischaemia injury is increasingly recognized to substantially affect quality of life, morbidity, and survival after heart transplantation [2]. Alleviating RA induced by hypothermia-ischaemia has become the most important consideration for heart transplant patients.

Gap junctions (GJs), mainly formed by connexin 43 (Cx43), allow chemical and electric coupling between cardiomyocytes [3]. GJ remodelling is well established as a consistent feature in human heart disease involving spontaneous ventricular arrhythmias [4]. Some studies have indicated that alterations in the distribution and expression of Cx43 are dominant factors that cause GJ remodelling, slowing conduction velocity (CV) and increasing transmural repolarization dispersion (TDR). In the presence of pathophysiology, these abovementioned changes also contribute to the formation of an arrhythmogenic substrate [5, 6].

There is abundant evidence that protein kinase C (PKC) phosphorylates Cx43 at serine 368 (Cx43-pS368), which can increase GJ uncoupling in addition to promoting Cx43 endocytosis or internalization and degradation [7, 8]. In addition, Cx43-pS368 can clearly effectively restrict ischaemia-reperfusion injury and GJ remodelling [9, 10].

Sevoflurane, a commonly used volatile anaesthetic, can attenuate ischaemia-reperfusion-induced ventricular arrhythmias [11]. Previous studies have demonstrated that sevoflurane alleviates epinephrine-induced arrhythmias in rats after brain death, an effect that may be related to the cardiac gap junction channel protein Cx43 [12]. In addition, sevoflurane may have an inhibitory effect on GJs and is known to play a cardioprotective role [13–15]. Some drugs that have the same effect as sevoflurane can also be used to suppress GJ activity to reduce cardiac infarct size in animal models [16]. As mentioned above, we tested the hypothesis that sevoflurane, as an additive to HTK solution will attenuate RA through reduced gap junction remodelling via the phosphorylation of Cx43 at serine 368 in denervated transplanted hearts after prolonged hypothermic ischaemic storage.

This work aimed to explore the potential mechanisms by which sevoflurane, as an additive to HTK solution, reduces RA. Therefore, changes in CV were recorded by using an S1S2 protocol for programmed electrical stimulation (PES) during monophasic action potential (MAP) recording. The total Cx43 level, Cx43-pS368 and Cx43 distribution were assessed, and the Cx43-pS368/total Cx43 ratio and TDR, as well as myocardial ischaemia-reperfusion injury and cell apoptosis, were sufficiently investigated.

## Methods

### Ethical approval

The study conformed to the Guide for the Care and Use of Laboratory Animals published by the US National Institutes of Health (NIH Publication No. 85–23, revised 1996). All protocols were approved by the Ethics Committee of Guizhou Medical University (No.1800452).

### Preparation of solutions and isolation of mouse hearts

The preparation of the hearts as well as the Langendorff-setup has been previously described in detail [17]. The Krebs-Henseleit (KH) solution contained (in mM): 118 NaCl, 4.7 KCl, 1.2 MgSO_4_7H_2_O, 1.2 KH_2_PO_4_, 25 NaHCO_3_, 11 glucose, and 10 HEPES (pH = 7.35, adjusted with NaOH). The solution was passed through a 22 μm filter before use. For Langendorff perfusion, the KH solution was equilibrated with 95%O_2_–5%CO_2_.Histidine-tryptophan-ketoglutarate (HTK) solution were purchased from Sigma-Aldrich (St. Louis, MO, USA). The HTK solution was aerated with 2.5% sevoflurane using a Vapor 2000 for at least 15 min. The concentration of sevoflurane was measured using a high-performance liquid chromatography (Thermo Scientific UltiMate 3000 HPLC, Massachusetts, USA). All doses were similar to those administered in previous experimental investigations and in clinical studies.

### Prolonged hypothermia-ischaemia-reperfusion protocol and groups

The hearts were divided into two groups according to a random number table: all hearts were arrested by an infusion of HTK solution (4 °C), followed by (i) storage in HTK solution (4 °C) alone for 6 h (*n* = 8, Control group) or (ii) storage in HTK solution supplemented with sevoflurane (4 °C) for 6 h (n = 8, Sevo-HTK group) [18]. Subsequently, the hearts were remounted on the perfusion apparatus and reperfused in Langendorff mode for 45 min. Hearts with a baseline heart rate (HR) of less than 180 beats/min were excluded at this stage.

### Electrophysiological measurements and ECG monitoring

MAP duration (MAPD) recordings [19] from the left ventricular (LV) epicardium and endocardium were obtained using a custom-made MAP electrode that was made from two strands of 0.25 mm silver wire. A reference electrode was immersed in the KH solution surrounding the heart. The MAP was amplified and analysed using a BL-420 biological function system. Bipolar electrograms between the atrial and ventricular electrodes were used to monitor ventricular arrhythmias [20] (Chengdu Tai League Software Co., Ltd., Chengdu, China).

### Stimulation protocols

Paired platinum electrodes (5 mm interpolar distance) were used to electrically stimulate the LV epicardium at 8 Hz, using square wave pulses of 2 ms and a stimulation voltage of three times the diastolic threshold (Suzhou Electronic Instrument Factory, Suzhou, China) after reperfusion for 45 min. The S1S2 protocol was used to assess arrhythmogenicity and identify reentrant substrates. This protocol consisted of a drive train of eight regularly paced S1 stimuli separated by a 125 ms basic cycle length (BCL), followed by premature S2 extra-stimuli every ninth stimulus. The S1S2 interval was first set to 125 ms and was then successively reduced by 1 ms every ninth stimulus cycle until arrhythmic activity was initiated or refractoriness occurred, in which the S2 stimulus elicited no ventricular response [21].

### Western blotting

Total protein (100 μg) was separated on 12% SDS-polyacrylamide gels and electrotransferred to nitrocellulose membranes. The membranes were incubated overnight with the primary antibody (anti-Cx43, 1:1000; anti-Cx43-pSer368, 1:1000). After incubation with the horseradish peroxidase-conjugated secondary antibody (GAPDH, 1:5000), the relative levels of the target proteins were determined by chemiluminescence [22].

### Immunohistochemistry

Micro-patterned monolayers were washed with cold PBS 3 times and were then fixed with methanol at − 20 °C for 15 min. The monolayers were then blocked with PBS/1% BSA, 1% normal goat serum, and 0.1% Triton X for 30 min at room temperature. The monolayers were probed for total Cx43 (above) overnight at 4 °C. Following 3 washes with PBS, Cx43 was detected with an anti-rabbit secondary antibody for 1 h at room temperature. The monolayers were washed 3× in PBS and mounted with mounting media (Guizhou Medical University Laboratories). Laser scanning confocal microscopy was used to obtain images, which were analysed for signal location [23].

### HE and TUNEL staining

A terminal deoxynucleotidyl transferase-dUTP nick end labelling (TUNEL) assay was conducted using a commercial kit according to the manufacturer’s instructions (C1086, Wuhan BIOFAVOR Company, China). Generally, 10 μm thick slices of myocardial tissue from each group were fixed with 4% paraformaldehyde for 30 min and permeabilized with 0.5% Triton X-100 for 5 min. Next, the TUNEL reaction mixture was added into each slide and incubated in a humidified chamber at 37 °C for 60 min. The results of the TUNEL reaction were observed under an inverted fluorescence microscope (Leica DMI8) with excitation and emission wavelengths of 495 nm and 519 nm, respectively. The images from each group were obtained using a digital CCD camera (Leica DFC450). We qualitatively analysed myocardial apoptosis by TUNEL staining as previously described [24]. In addition, haematoxylin and eosin (HE) staining was performed in accordance with previously reported protocols [25].

### Statistical analysis

All data are presented as the means ± standard errors of the mean. The incidence was compared with Fisher’s exact test, and different experimental groups were compared by Student’s t test and analysis of variance using SPSS 22.0 (IBM SPSS Statistics, USA). *P* < 0.05 was considered to indicate a statistically significant difference between the Sevo-HK and Control groups.

## Results

### Sevo-HTK solution decreases RA

**Fig. 1:**
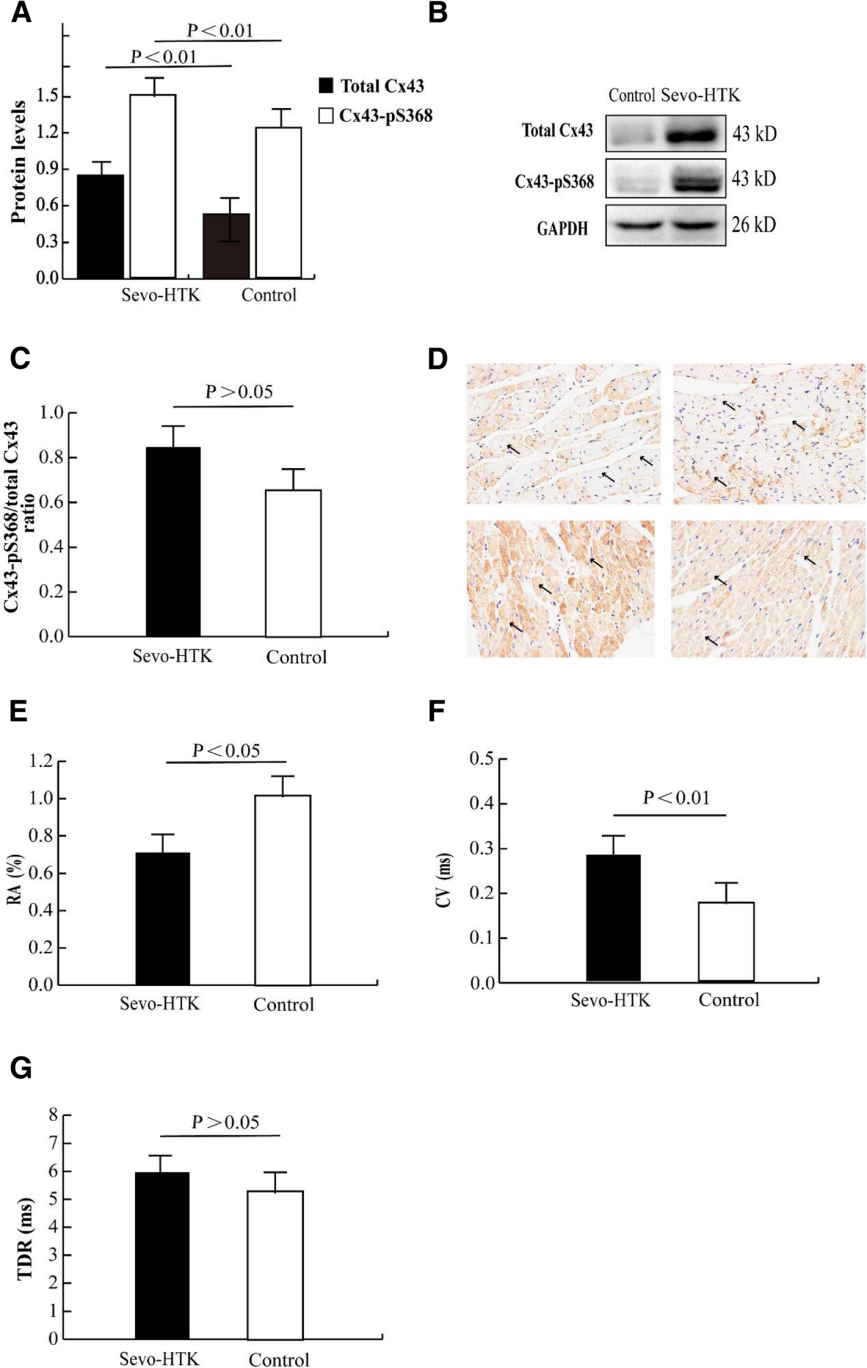
Sevoflurane, as an additive to HTK solution, prevented gap junction Remodelling and electrophysiological properties promoting arrhythmogenesis, and also resulted in Cx43 phosphorylation at Ser368. **a** Quantitative analysis of the Western blotting results for Cx43 and Cx43-pS386 levels. **b** Western blot analysis of total Cx43 and Cx43-pS386 levels. **c** Ratio of total Cx43 to Cx43-pS386. **d** Cx43 distribution via immunohistochemical methods (400 ×). The black arrow indicates the brownish- red granular material as the Cx43 protein. The two upper images, show heterogeneous distribution and lateralization of Cx43 in the Control group. The two lower images show homogeneous distribution and stabilization of Cx43 in the Sevo-HTK group. **e** Difference in the RA between the groups. **f** Difference in the conduction velocities. **g** Similar TDR in the two groups

RA was successively observed in 8/8 hearts stored in HTK alone and in 5/8 hearts stored in HTK supplemented with sevoflurane. (Fig. 1e).

### Sevo-HTK solution reduces GJ remodelling and promotes Cx43 phosphorylation at serine 368

Sevo-HTK solution increased the levels of total Cx43 (1.59 ± 0.59 vs 0.85 ± 0.21, *P*<0.01) and Cx43-pS368 (1.23 ± 0.54 vs 0.53 ± 0.27, *P*<0.01) (Fig. 1a and b) and prevented Cx43 redistribution (including Cx43 lateralization and heterogeneity) (Fig. 1d). The Cx43-pS368/total Cx43 ratio was similar (0.66 ± 0.32 vs 0.84 ± 0.37, *P* > 0.05) (Fig. 1c) and the Cx43-pS368 levels were lower than the total Cx43 levels in both the Control and Sevo-HTK groups (Fig. 1a).

### Sevo-HTK solution prevents CV slowing but does not change TDR

As an additive to HTK solution, sevoflurane prevented CV slowing (0.29 ± 0.02 vs 0.18 ± 0.02 ms, *P*<0.001) (Fig. 1f) but did not change the TDR relative to that in the Control group (5.22 ± 3.04 vs 6.27 ± 3.03 ms, *P*>0.05) (Fig. 1g).

### Sevo-HTK solution ameliorates myocardial ischaemia-reperfusion injury

**Fig. 2:**
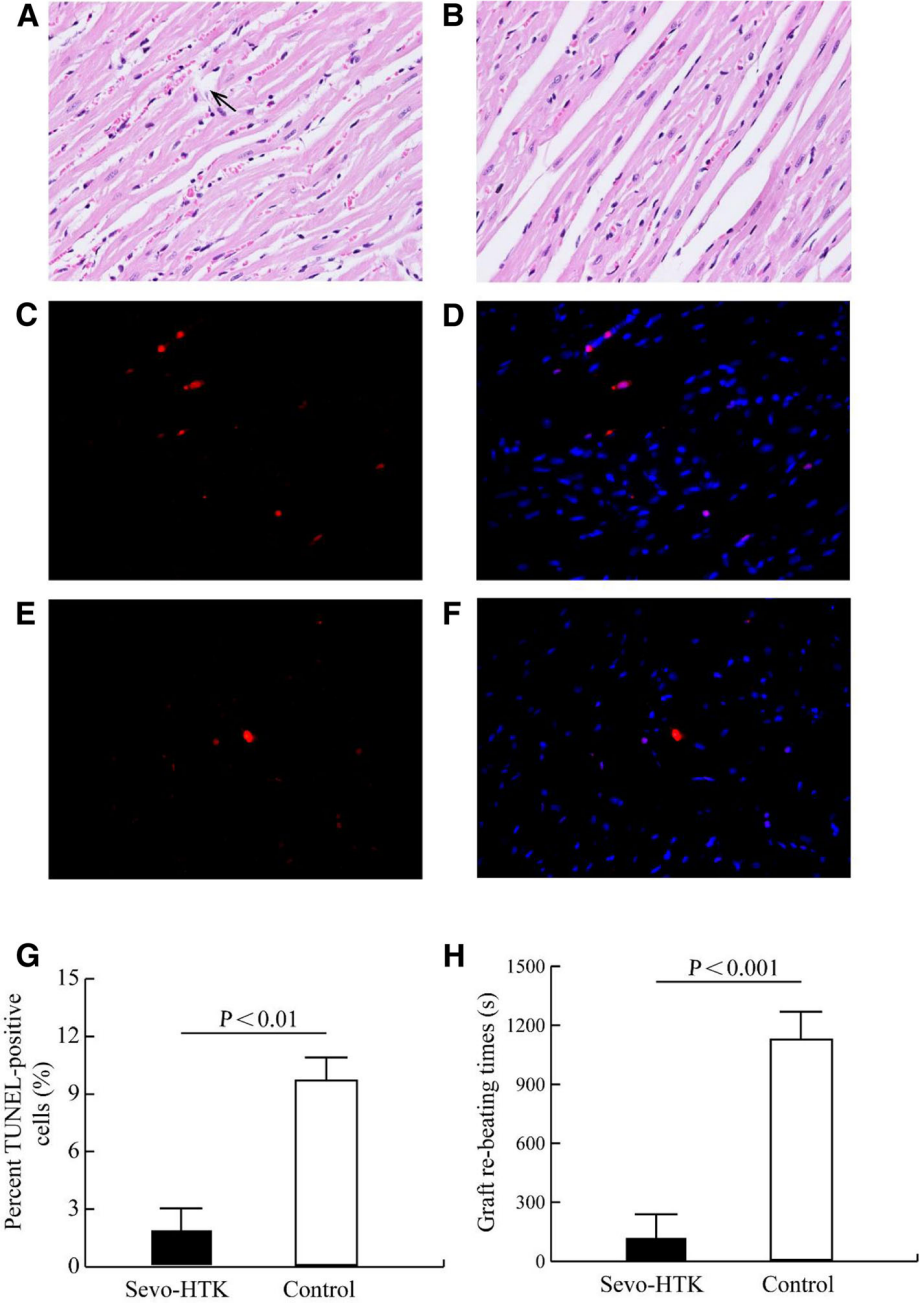
HE and TUNEL staining in myocardial tissue (400×). Apoptotic nuclei were identified by TUNEL staining (red), and total nuclei were identified by DAPI counterstaining (blue). **a** Part of the cardiac muscle fibres appeared broken, swollen and disordered; the black arrow indicates fractured cardiac muscle fibres in the control. **b** Pathological damage to the myocardium was ameliorated in donor hearts stored in Sevo-HTK solution. **c** and **d** Show that the number of apoptotic nuclei increased in the Control group. **e** and **f** Show that the number of apoptotic nuclei decreased in the Sevo-HTK group. **g** Sevoflurane as an additive appreciably lowered the number of apoptotic cells. **h** Difference between the graft re-beating times in the two groups

HE staining showed that the myocardial pathological damage was ameliorated in hearts stored in Sevo-HTK solution, and TUNEL staining showed that the number of apoptotic cells was markedly reduced (9.5 ± 0.5% vs 2.3 ± 0.3%, P<0.01). Sevo-HTK solution also appreciably shortened the graft re-beating time (1150 ± 51 vs 51 ± 5 s, P<0.001) (Fig. 2).

## Discussion

Cx43 is the major component of ventricular GJs, and alterations in Cx43 expression and distribution can induce ventricular arrhythmias [26]. Particularly, the expression and heterogeneous distribution of Cx43 (for example, Cx43 lateralization) was decreased [27]. Regardless of arrhythmogenesis induced by ischaemia or hypothermia, it can be attenuated by preventing GJ remodelling [28, 29]. We found a homogeneity and stabilization of Cx43 distribution in the Sevo-HTK group, accompanied by the alleviation of RA, while heterogeneity and lateralization of the Cx43 distribution were found in the Control group, which exhibited a high rate of RA (Fig. 1d and e). This observation is consistent with the previous hypothesis that Sevo-HTK solution reduces GJ remodelling and thus reduces RA. Thus, Sevo-HTK solution attenuates ventricular arrhythmogenicity in denervated transplanted hearts during reperfusion and is possibly connected with preventing Cx43 degradation and redistribution.

GJs that produce low-resistance pathways between cardiomyocytes are major determinants of electrical conduction in the heart [30]. Electrical uncoupling at GJs in the diseased heart contributes to conduction abnormalities or repolarization heterogeneity for reentrant arrhythmias [31], highlighting the importance of Cx43 in maintaining proper conduction. We regarded TDR as a special marker of an abnormality in cardiac electrical repolarization; TDR has high accuracy in risk prediction, and MAPD variations between different cardiac regions (maximal MAPD – minimal MAPD) can also be observed. Exacerbated variations (for example, increased TDR) can predispose to reentrant arrhythmia from arrhythmogenesis [32]. In general, dispersion of repolarization promotes susceptibility to reentrant arrhythmias. There is at least 1 mechanism by which reduced gap junction coupling can enhance TDR. Although electrical uncoupling between cells increases differences in MAPD between cells with different ionic compositions across the heart by decreasing the electrotonic current flow between neighbouring cells, we observed that the value of TDR was similar in both study groups (Fig. 1g). Additionally, different treatment doses of sevoflurane that did not change TDR have been widely reported in human and animal models, and is the idea that TDR is a poor predictor of arrhythmia has been verified by other models [33]. Gary Tse found a contradiction in his study indicating that increased TDR does not correspond to the development of RA, particularly in models of long QT syndrome (LQTS) and short QT syndrome (SQTS) [34]. A reduction in the expression and redistribution of Cx43, which leads to intercellular uncoupling, is directly correlated with anisotropic conduction slowing, thus increasing the propensity for arrhythmogenesis. In contrast, the preservation of intercellular coupling, which could prevent conduction slowing, eliminated arrhythmogenic substrates [35]. Upregulating and reducing the redistribution of Cx43 may aid in improving conduction safety and protecting the myocardium against life-threatening tachyarrhythmias, especially in the presence of hypothermia or ischaemia. The present observation that Sevo-HTK solution inhibited Cx43 degradation and redistribution indicates that this solution is more likely than HTK alone to prevent conduction slowing (Fig. 1a and d, e and f).

PKC-mediated increases in Cx43 phosphorylation at serine 368 may contribute to reduced gap junction remodelling and aid in reducing inducible arrhythmia following injury. Previous research showed that localized treatment of LV injuries with αCT1 resulted in a decreased propensity to develop arrhythmia in response to programmed stimulation and ischaemic injury, which was attributed to αCT1 inducing an increase in PKC-mediated Cx43-pS368 levels [36]. Our observation of increased Cx43-pS368 levels was in accordance with Cx43 preservation and the relatively increased CV following injury, which may in turn explain the decreases in RA in hearts stored in Sevo-HTK solutions (Fig. 1a and d). However, similar observations have been made by others in ischaemia models. For example, Kardami and colleagues showed that ischaemic preconditioning (IPC) or FGF-2 treatment increased the PKC-mediated phosphorylation of serines at positions 262 and 368 in Cx43 and inhibited the remodelling of Cx43 in response to ischaemic insult [37].

Another mechanism in Cx43-pS368 phosphorylation has previously been suggested to be involved in the increased internalization and degradation of Cx43, which promotes GJ uncoupling mediated by relative increases in the relative level of Cx43-pS368 to that of total Cx43 [38–40]. In addition, a few drugs that can block gap junction communication reduced cardiac infarct size in animal models during reperfusion [41]. Our data showed that the level of Cx43-pS368 was lower than that of total Cx43 and that the Cx43-pS368 phosphorylation/total Cx43 ratio was similar in both groups (P>0.05) (Fig. 1a and c). A previous study reported that inhibiting GJs may be a function of sevoflurane [42]. Thus, sevoflurane, as an additive to HTK solution, could be associated with GJ uncoupling, but we have not proven this association in this work.

The phosphorylation of Cx43 at serine 368, which promotes a cardiac injury-resistant state, has cardioprotective effects [22]. The graft re-beating time, which is the time from reperfusion opening to the recovery of spontaneous rhythm and can reflect the severity of ischaemic injury or haemodynamic change, also indirectly reflects cardiac function [43]. We found that the donor hearts stored in different heart preservation solutions showed statistically significant differences in the graft re-beating times (Fig. 2h). The graft re-beating times after reperfusion for the hearts stored in HTK containing 2.5% sevoflurane were clearly shortened, possibly due to the prevention of severe ischaemia injury in the donor hearts. Yang and colleagues found that sevoflurane postconditioning confers myocardial protective effects similar to those of IPC and reduces ischaemia-reperfusion injury [44]. Coincidentally, our HE staining results also showed that the degree of myocardial pathological damage was significantly ameliorated in donor hearts stored in Sevo-HTK solution (Fig. 2a and b), and TUNEL staining showed that sevoflurane as an additive caused a pronounced decrease in the number of apoptotic cells (Fig. 2c, d, e, f and g). These data also supported the outcome of alleviating ischaemia-reperfusion injury. Ischaemia-reperfusion injury is strongly associated with GJ remodelling, and preventing the decrease and redistribution of Cx43 through ischaemic postconditioning could protect the heart from ischaemia-reperfusion injury. As previously described, the donor hearts stored in Sevo-HTK exhibited less injury than the control hearts, which could be strongly linked to the prevention of GJ remodelling. Indeed, Cx43-pS368 has been defined as a ‘marker’ for the development of an injury-resistant state, and its cardioprotective effects have been well established in many studies [45]. As mentioned earlier, Cx43-pS368 was also sufficiently proven to be associated with reduced GJ remodelling in this study.

## Conclusion

The reduction in hypothermia and ischaemia-induced reperfusion arrhythmias by the addition of sevoflurane to HTK solution may be related to the phosphorylation of Cx43 at serine 368.

## Abbreviations

BCL: Basic cycle length
CV: Conduction velocity
Cx43: Connexin 43
Cx43-pS368: Cx43 phosphorylated at Ser368
ECG: Electrocardiography
GJs: Gap junctions
HE: Haematoxylin and eosin
HTK: Histidine-tryptophan-ketoglutarate
Hz: Hertz
IPS: Ischaemic preconditioning
KH: Krebs-Henseleit
LQTS: Long QT syndrome
MAP D_90_: Monophasic action potential at a repolarization of 90%
MAP: Monophasic action potential
MAPD: Monophasic action potential duration
ms: Milliseconds
mV: Millivolts
PES: Programmed electrical stimulation
PKC: Protein kinase C
PLC: Pacing cycle length
RA: Reperfusion ventricular arrhythmia
SPSS: Statistical Package for Social Science
SQTS: Short QT syndrome
TDR: Transmural repolarization dispersion
TUNEL: Terminal deoxynucleotidyl transferase-dUTP nick end labelling
